# Impact of Pharmacists’ Intervention on the knowledge of HIV infected patients in a public sector hospital of KwaZulu-Natal

**DOI:** 10.4102/phcfm.v3i1.258

**Published:** 2011-10-12

**Authors:** Saloshini Govender, Tonya Esterhuizen, Panjasaram (Vassie) Naidoo

**Affiliations:** 1 School of Pharmacy and Pharmacology, University of KwaZulu-Natal, South Africa; 2 Programme of Biostatistics, Research Ethics and Medical Law, College of Health Sciences, South Africa

## Abstract

**Background:**

The study site started its roll-out of the human immunodeficiency virus (HIV) prevention of mother-to-child transmission in 2006. All patients were counselled by trained counsellors, before seeing a doctor. At the pharmacy the medicines were collected with no intense counselling by a pharmacist as the patients would have visited the trained counsellors first. Subsequently it was found that there were many queries regarding HIV and acquired immune deficiency syndrome (AIDS). Thus a dedicated antiretroviral pharmacy managed by a pharmacist was established to support the counsellors.

**Objectives:**

The objective of the study was to assess the impact of a pharmacist intervention on the knowledge gained by HIV and AIDS patients with regard to the disease, antiretroviral drug use (i.e. how the medication is taken, its storage and the management of side effects) as well as adherence to treatment.

**Method:**

This study was undertaken at a public sector hospital using anonymous structured questionnaires and was divided into three phases: pre-intervention, intervention and post-intervention phases. After obtaining patient consent the questionnaires were administered during the first phase. A month later all patients who visited the pharmacy were counselled intensely on various aspects of HIV and antiretroviral medication. Thereafter patients who participated in Phase 1 were asked to participate in the second phase. After obtaining their consent again, the same questionnaire was administered to them. Quantitative variables were compared between pre-intervention and post-intervention stages by using paired t-tests or Wilcoxon signed ranks tests. Categorical variables were compared using McNemar's Chi-square test (Binary) or McNemar-Bowker test for ordinal variables.

**Results:**

Overall the mean knowledge score on the disease itself had increased significantly (s.d. 6.6%), (*p* < 0.01), after the pharmacists’ intervention (pre-intervention was 82.1% and post-intervention was 86.3%). A significant improvement was noted in the overall knowledge score with regard to medicine taking and storage (*p* < 0.05) and the management of the side effects. There was a non-significant difference between the adherence in pre-intervention and in post-intervention (*p* = 0.077).

**Conclusion:**

Pharmacists’ intervention had a positive impact on HIV infected patients’ HIV and AIDS knowledge on both the disease and on the antiretroviral drug use and storage.

## Introduction

### Setting

#### Key focus

Human immunodeficiency virus (HIV) remains a global health problem of unprecedented dimensions. Three decades ago this virus was unknown; yet it has already caused an estimated 25 million deaths worldwide and has generated profound demographic changes in the most heavily affected countries.^1^

In the countries that are most heavily affected, HIV has reduced life expectancy by more than 20 years, slowed economic growth, and deepened household poverty. In sub-Saharan Africa alone, the epidemic has orphaned nearly 12 million children under the age of 18. The natural age distribution in many national populations in sub-Saharan Africa has been skewed dramatically by HIV.^1^

Sub-Saharan Africa remains the region most heavily affected by HIV, accounting for 67% of all people living with HIV and for 75% of acquired immune deficiency syndrome (AIDS) related deaths in 2007.^1^

In Southern Africa, South Africa is home to the world's largest population of people living with HIV (5.7 million).^1^ The prevalence of HIV in South Africa varies by province, with KwaZulu-Natal having the highest prevalence with 15.8% of people affected by HIV.^2^

The South African Government's response to the HIV and AIDS epidemic is found in the HIV and AIDS and STI Strategic Plan for South Africa of 2007–2011. The plan purposed to provide a broad national framework around four areas:

- prevention and treatment
- care and support
- research, monitoring and evaluation
- human and legal rights.^3^

HIV is present in the blood and genital secretions of virtually all infected individuals regardless of whether or not they have symptoms. The spread of HIV can occur when these secretions come into contact with tissues such as those lining the vagina, the anal area, mouth, or eyes, or a break in the skin, such as a laceration or a puncture by a needle.^4^ The transmission of HIV is normally via sexual contact, needle sharing, and transmission from infected mothers to their babies during pregnancy, labour or breast-feeding. Sexual transmission of HIV has been described from men to men, men to women, women to men and women to women through vaginal, anal and oral sex. The ideal to avoid sexual transmission would be abstinence from sex until it is certain that both partners, who are in a monogamous relationship, are not HIV infected. If abstinence is not possible then the use of condoms is encouraged. The spread of HIV by exposure to infected blood usually results from sharing needles, as in those used for illicit drugs. Unless there are open sores or blood in the mouth, kissing is generally considered not to be a risk factor for transmitting HIV.^4^

The mother-to-child transmission rate had increased at a phenomenally high percentage. Whereas in 1990, the HIV prevalence amongst pregnant women attending antenatal clinics in South Africa was less than 1%, the prevalence had risen 10 years later, in the year 2000, to 24.5% causing an estimated 550 000 children to be infected with the virus. In many low-resource settings, the most feasible regimen was intra-partum and post-partum Nevirapine, for reasons of cost and convenience. Nevirapine was advocated to be given to the mother as soon as possible after the onset of labour. Nevirapine decreased the incidence of transmission of HIV and AIDS from mother to child by 50%–70%.^5^ The introduction of HAART ((highly active antiretroviral therapy) had revolutionised the treatment of HIV and AIDS patients with regard to the management of HIV infected persons. These effectively inhibit viral replication, prevent drug resistance and prevent immune dysfunction. Antiretroviral drugs are arranged in five classes:

- nucleoside or nucleotide reverse transcriptase inhibitors (NRTIs)
- non-nucleoside reverse transcriptase inhibitors (NNRTIs)
- protease inhibitors (PIs)
- entry inhibitors
- integrase inhibitors.^6^

The HAART regimens comprise at least three drugs and require that numerous drugs be taken every day to achieve maximum viral suppression.^7^ Adherence to these drugs by HIV patients is therefore vital in order to achieve optimum therapeutic outcomes. Antiretroviral therapy can fail because of poor adherence, acquired drug resistance, poor drug absorption, drug-drug interactions or lack of drug potency.^8^ Failure to take all of the prescribed doses of antiretroviral medication may result in inadequate drug concentrations, which can result in incomplete inhibition of HIV replication and may accelerate viral resistance to antiretroviral therapy.^9^

Antiretroviral medication must be stored correctly in order to remain potent and to have the desired pharmacological effect. Drugs such as Lamivudine, Zidovudine, Stavudine, Nevirapine and Efavirenz should be stored below 25°C in a cool, dry place and should be protected from moisture and light, whilst Kaletra^®^ is stable until the expiry date if refrigerated or kept for 2 months at room temperature.^10^

The public sector roll-out programme in South Africa for the prevention of mother-to-child transmission with Nevirapine started at 18 pilot sites in 2000. An antiretroviral (ARV) treatment plan was published in 2003 and the roll-out of antiretroviral treatment started at 32 accredited sites in April 2004, aiming to treat all South Africans that needed therapy.^11^

Some drugs cannot be given concomitantly; many drugs alter the concentration of others, resulting in toxic and sub-therapeutic effects, thereby demanding a change in dosage of one or more medications. Some oral medication should be given with food^12^ or cannot be taken with certain foods, some on an empty stomach, and many have to be taken on a very specific schedule so that the drug's concentration in the bloodstream does not vary.^13^ Some medication should not be co-administered with other medication whilst certain medication will cause therapeutic failure.^12^ It is the responsibility of the pharmacist to ensure that the patient and caregivers are aware of these dietary restrictions.^14^

The ARV clinic at the study site was in operation since October 2006. Prior to the study conducted, all patients were counselled by trained counsellors on three consecutive visits to the ARV clinic, before being seen by a doctor for the first time. Then the patients would go to the ARV pharmacy where pharmacists would hand over the medication, explaining only when and how often patients have to take the medication. No intense counselling on the ARV medication was conducted.

Subsequently it was found that there were many queries from patients attending the HIV clinic regarding knowledge on the disease, how it is transmitted, and the measures that can be taken to prevent HIV infection. Other queries related to the medication, its side effects, storage conditions and so forth. A decision was made to establish the ARV pharmacy and to have it managed by a dedicated ARV pharmacist who would support the counsellors by reinforcing what was said by counsellors, as well as giving detailed information on their disease condition, HIV prevention methods, medication, side effects, and medication storage.

Pharmacists are often considered the most accessible health professional^14^ whose core function is the provision of pharmaceutical care, in essence the responsible provision of drug therapy for the purpose of achieving definite outcomes which improve the patients’ quality of life.^15^ Overall, the pharmacists’ responsibility is to optimise a patient's medication therapy.^14^ Amongst the many medical conditions that pharmacists deal with are HIV and AIDS.

As of from May 2007, pharmacists were expected to dispense ARV medication and counsel the patients. This study was subsequently undertaken to assess the impact of a pharmacist's intervention on the knowledge gained by HIV and AIDS patients with regard to the disease, antiretroviral drug use (that is, how the medication is taken, its storage and the management of side effects) as well as adherence to treatment.

## Ethical consideration

Ethical approval to conduct this study was obtained from the University of KwaZulu-Natal Research and Ethics Committee (Ethics Number: FECHSC 002/07). Permission to administer the questionnaire to patients was obtained from the senior management of the study site and the Provincial Department of Health in KwaZulu-Natal. Anonymity of responses was maintained by requesting participants not to disclose their personal details on the questionnaires.

## Method

### Setting and material

The study was undertaken at a public sector hospital (Community Health Centre) at which the principal investigator was based during October 2007 and November 2007. This Community Health Centre is situated in the Northern Suburb of the eThekwini Metro of KwaZulu-Natal. The HIV clinic at the study site sees people of different cultures, religious denominations and races. All HIV infected patients who visited the clinic during the time period of October 2007 to November 2007 were invited to participate in the study, in total a number of 400 patients.

### Design

This was an intervention study divided into three phases: a Pre-intervention Phase (Phase 1), an Intervention Phase (Phase 2), and a Post-intervention Phase (Phase 3). There was no control group as participants acted as their own controls in the pre-intervention phase.

#### Pre-intervention Phase (Phase 1)

During their clinic visit in the study period, the study was explained to each participant and a consent form was completed. A pre-tested questionnaire which was anonymous and structured was handed to all participants in their language of choice. The questionnaire included sections on demographics, knowledge of medicines, the taking of medicine and disease conditions together with questions on storage, side effects experienced and adherence patterns.

On completion of the questionnaires, they were collected and coded. A register of these participants with their corresponding coding was opened and their details entered in the register.

#### Intervention Phase (Phase 2)

At the second visit, one month after Phase 1, all patients who visited the clinic were counselled by the pharmacist on all aspects of their medicines, medicine taking, and disease condition, together with information on storage, side effects experienced and the importance of adherence by helping patients plan dose times, explaining what to do if a dose was missed and suggesting ways to remember to take medication, as well as managing side effects.

Participants who took part in Phase 1 of the study were once again asked for consent to participate in Phase 2 of the study.

#### Post-Intervention phase (Phase 3)

The same questionnaire that was used in Phase 1 was filled out again by the participants who consented to be part of Phase 2. The register of the participants was destroyed after the completion of Phase 3 of the study in order to ensure confidentiality.

### Analysing

The data were captured and analysed using SPSS (Statistical Package for the Social Sciences) version 15.0 (SPSS Inc., Chicago, Illinois, USA). Knowledge was scored by allocating a score of 1 to each correct answer, summing up the scores, and subsequently expressing the scores as a percentage of the total possible score with higher percentages indicating higher levels of knowledge. The changes in all factors between pre-intervention and post-intervention were assessed statistically. Quantitative variables were compared between pre-stages and post-stages using paired t–tests or Wilcoxon signed ranks tests. Categorical variables were tested using McNemar's Chi-square test (Binary) or the McNemar-Bowker test for ordinal variables.

## Results

### Response Rate

Two-hundred (50%) patients consented to be part of Phase 1 of the study. In Phase 2, one hundred and seventy five patients completed the questionnaire. Thus 175 participants completed both phases of the study, resulting in a response rate of 87.5%.

The demographic profile of the participants is depicted in Table 1.

**TABLE 1:** Demographic profile of participants (*N* = 175).

Demographic profile	*n*	%
**Gender**		
Male	39	22.3
Female	136	77.7
**Age Range**		
21–30	47	26.86
31–40	75	42.86
41–50	45	25.71
51–60	7	4. 00
61–70	1	0. 57
**Post-school Education**		
Yes	13	7.4
No	162	92.6

*Source:* Authors’ original data *n*, Number of participants.

The majority of the patients were female. Almost 70% of the participants were in the age-range of 21–40 years old.

The majority of the participants did not have post-school education.

### Respondents’ knowledge of HIV and AIDS

#### General HIV and AIDS knowledge

The general knowledge of the participants had increased with all but one question after the pharmacist's intervention.

The question on whether HIV is a bacterium that causes AIDS was answered incorrectly by the majority of the participants (95.4%); however, there was a slight improvement with correct answers during the post-intervention phase.

The participants’ knowledge of whether people who have sexually transmitted diseases are least at risk of contracting the HIV virus, healthy food will cure HIV, and whether smoking and drinking alcohol will weaken the HIV virus, increased significantly from the pre-intervention phase to the post-intervention phase.

#### Knowledge on modes of transmission

The participants’ knowledge on the modes of transmission either increased or remained unchanged (Table 2 and 3). The majority of participants (94.6%) did not know that sexual contact with a homosexual with an unknown HIV status could result in a HIV infection.

**TABLE 2:** Respondents’ general knowledge of HIV and AIDS (*N* = 175) for Pre-intervention and Post-intervention.

Question	Intervention %	

	Pre	Post
HIV is a bacterium which causes AIDS	4.6	6.3
People with AIDS cannot fight sicknesses such as diarrhoea, TB and pneumonia	61.7	46.3
The HIV virus attacks the heart and liver	49.1	53.1
You can tell from a person's appearance that they have the HIV virus	80.5	91.4
A person has less chance of being infected with the HIV virus if they have one partner	84.0	89.7
A person can become infected with the HIV virus if they have unprotected sex with someone who has HIV	97.1	98.9
People who have sexually transmitted infections (STIs) are the least at risk of becoming infected with the HIV virus	35.4	61.1
Not all babies whose mothers are HIV positive, are born with the virus	89.7	91.9
Healthy food will cure HIV	52.9	86.3
Smoking and drinking alcohol will strengthen the body	98.3	98.3
Smoking and drinking alcohol will weaken the HIV virus	68.0	93.1
Support groups cannot help people with HIV and AIDS to deal with anger and loneliness	80.6	93.7
Diarrhoea is when you pass two or more loose or watery stools a day	93.1	94.9
When a person starts to cough, cough mixtures to stop the cough can be taken	63.4	84.0
People with HIV and AIDS have problems with their skin, for example rash	97.1	98.3
A person can be re-infected with HIV	92.6	95.4
Antiretroviral medication is a complete cure for HIV and AIDS	87.4	88.6

*Source*: Authors’ original data *N*, Number of participants; TB, Tuberculosis.

**TABLE 3:** Respondents’ knowledge of the modes of transmission: Pre-transmission and Post-transmission (*N* = 175).

Modes of Transmission	Intervention %	

	Pre	Post
Sharing needles with a person infected with HIV and AIDS	97.7	99.4
Unprotected sexual contact with a person infected with HIV and AIDS	99.4	100.0
Contact with blood	98.3	98.3
Sharing toilet seats with a person infected with HIV and AIDS	93.1	95.4
Using the same eating and drinking utensils of a person infected with HIV and AIDS	95.4	96.0
Kissing a person infected with HIV and AIDS who has sores in his or her mouth	84.0	87.4
Touching a HIV patient	94.9	94.9
Being bitten by a mosquito	68.6	75.4
Sexual contact with a homosexual with an unknown HIV status	15.4	15.4
Working with a person infected with HIV and AIDS	96.6	96.0
Sexual contact with too many partners with an unknown HIV status	92.0	97.1

*Source*: Authors’ original data *N*, Number of participants.

#### Respondents’ knowledge on HIV prevention

The overall mean knowledge score, Pre-intervention, was 82.1% with a standard deviation of 6.6%, whilst after the Intervention it had increased to 86.3% (SD 6.6%). This increase was statistically significant (Table 4 and 5).

**TABLE 4:** Respondents’ knowledge of HIV prevention; Pre-intervention and Post-intervention (*N* = 175).

HIV Prevention	Intervention %	

	Pre	Post
Abstinence from sex	93.7	94.9
The use of latex barriers (condoms)	97.7	97.1
Avoiding needle sharing	98.9	98.3
By being faithful to one partner	97.1	96.0
By not associating with HIV and AIDS patients	91.4	92.6

*Source*: Authors’ original data *N*, Number of participants.

**TABLE 5:** Paired *t*-test for the comparison of mean knowledge score Pre-intervention and Post-intervention.

Paired *t*-test	Mean	*N*	*STI* Deviation	*STI* Error Mean	*p-v*alue
knowledge score Pre-intervention	82.10	175	6.598	0.499	*<* 0.001*
knowledge score Post-intervention	86.35	175	6.628	0.501	-

*Source:* Authors’ original data *N*, Given as means of Total number; *STI*, Sexual Transmitted Infection.

* *p* = 0,000.

### Respondents knowledge on antiretroviral drug use

#### How medication is taken

The frequency of initially reporting taking Lamivudine, Stavudine and Efavirenz with a high fatty meal or any time they remembered decreased to zero after the Intervention. The majority of the patients took their medication either with food or without food at both phases of the study.

#### Respondents knowledge on storage of antiretroviral medication

In both phases, over 40% of all the patients stored their medication in a cupboard. There were no significant differences in terms of storage of the antiretroviral medication in both pre-intervention and post-intervention.

Some of the participants chose *Other* as their answer. *Other* referred to storage in the bedroom, drawers in the bedroom, a drawer in the dressing table, the headboard, in a box, in a bag, in a shoe box, in an underwear drawer, on top of a wardrobe and on shelves in the bedroom.

#### Management of side effects

When asked whether the side effects of the medication were explained to the participants, 98.3% (*n* = 172) said *yes* whilst 1.7% (*n* = 3) said *no* in the pre-intervention phase. This increased to 100% (*n* = 175) in the post-intervention phase.

In terms of participants’ understanding of medication and side effects, when they occur, 96% (*n* = 168) said they would go to a doctor or clinic immediately in the pre-intervention phase. This increased to 99.4% (*n* = 174) at post-intervention.

When asked what they would do if they vomited after taking their medication, 94.9% (*n* = 166) stated that they would take their medication again if they vomited less than 30 minutes after taking the medication, and at post-intervention this improved to 97.7% (*n* = 171). Furthermore, 92% (*n* = 161) said they would do nothing if they vomited more than 30 minutes after taking their medication pre-intervention whilst this improved to 96% post-intervention.

Overall the responses on drug storage, how the medication is taken, the management of side effects and what to do if vomiting occurs, were scored by assigning a score of 1 for each correct response. The maximum score was 5.

Respondents generally scored high, with the median score pre-intervention equal to 4 (inter-quartile range 4–5), whilst the median increased significantly to 5 (inter-quartile range 4–5) (*p* = 0.024) in the post-intervention phase.

### Adherence to treatment

Patients were counselled on adherence during the postintervention phase, but there was a non-significant difference in adherence pre-intervention and post-intervention (*p* = 0.077). Twelve participants improved their adherence from pre-intervention to post-intervention whilst in another four respondents the adherence to medicines worsened. Although this suggests an improvement trend, it could not be confirmed statistically.

## Discussion

The respondents’ knowledge on HIV and AIDS (*p* < 0.001) and on medicine taking and storage (*p* < 0.05) had increased significantly after the intervention of the pharmacist. This suggested that the pharmacist did make a positive impact on the knowledge of the HIV infected patients who attended the public sector facility where the study was carried out.

In terms of the specific questions asked on general knowledge, most of the participants knew about the disease with the exception of the question that related to HIV being a bacterium that causes AIDS, where a very small percentage knew the correct answer.

One possible reason could be that the participants may not have known that there is a difference between a bacterium and a virus as over 92% of them did not have post-school education.

The second possible reason, however, based on the fact that the pharmacist counselled the patients, could be that those respondents still believed that HIV (irrespective of whether it is a bacterium or a virus) does not cause AIDS. This belief could have been influenced by the dissident view shared by the previous South African President, Mr Thabo Mbeki, who stated that HIV was not responsible for AIDS.^16, 17^

With regard to sexually transmitted infections as a risk to contract HIV, just over a third of the respondents gave the correct answers. After the intervention, however, about two-thirds of the respondents displayed a greater knowledge about sexually transmitted infections and HIV infection. This finding is also supported by a study carried out in Ghana where it was found that pharmacists played a crucial role in the effective management of sexually transmitted infections.^18^

Another study carried out amongst high school students in Kathmandu Valley, found that pre-education knowledge of sexually transmitted infections was quite low (41.7%) compared to post-education knowledge (87.2%).^19^

Smoking and drinking alcohol were perceived by some of the respondents as a means of weakening the virus with only 68% of the respondents answering this question correctly before the intervention of the pharmacist. Even though this number greatly increased after the intervention, a small percentage (6.9%) still believed that smoking and drinking alcohol does have an effect on the virus. This could be one of the reasons why smoking and alcohol consumption are so common amongst HIV infected individuals as studies have shown.^20^ Other studies have shown that smoking and alcohol consumption amongst HIV positive individuals is high, attributing this to the stress caused by HIV status disclosure. Many patients also believed that to quit smoking would not improve their health.^21^

In this study a small percentage, both pre-intervention and post-intervention (12.6% and/or 11.4%), were of the opinion that AIDS could be cured. This correlated to a study conducted in Malaysia amongst young Malaysian adults where it was found that 18.1% believed that there was a cure for AIDS.^22^

A majority of the study population knew how HIV was transmitted with a further increase in the numbers knowing the different modes of transmission after the pharmacist's intervention. The percentage, however, that responded to the question on whether sexual contact with a homosexual with an unknown HIV status can cause AIDS, remained the same in both the pre-intervention and post-intervention phases, even though the pharmacist counselled them. A possible reason could be that the terminology ‘homosexual’ had been used and not the common terminology ‘gay’, which could lead to confusion amongst respondents as to the interpretation of the question, with an accompanying reluctance to asking for clarification. Some patients may feel embarrassed to ask questions. Studies have shown that patients are afraid to ask or request an explanation of terms or questions for fear of being ridiculed, fear of the illness and treatment or fear of the doctor; in this case it could be the researcher.^23^

Almost a third of the respondents believed that a mosquito bite could transmit the virus; however, of concern is that even after the intervention, a quarter of the respondents still maintained this belief. The belief that mosquitoes are vectors for HIV is a quite common misconception that has been shown in other studies as well.^24^

A further concern was that even though the respondents were counselled by the pharmacist and there was a positive outcome, there were still a small percentage of respondents that stuck to their own beliefs. With the exception of whether the act of unprotected sex can transmit the virus, the other responses in the post intervention phase did not give a 100% positive response to the questions. Of concern were questions on transmission (namely, via contact with blood, kissing of HIV infected persons with sores in their mouths, sexual contact with too many partners of unknown status could lead to risky behaviour) which implied that if respondents did not accept these means of possible modes of transmission, it could lead to a progression in the spread of the virus. Additional education on the different modes of transmission is consequently essential in order to prevent further transmission so that the epidemic can be contained.

Over 91% of the respondents were familiar with HIV prevention methods as they answered correctly to the questions given. A small percentage, however, still clung to the same views or beliefs even after the intervention. They maintained that abstinence from sex, the use of condoms, being faithful to one partner and so forth, would not contribute to HIV prevention methods. The finding relating to condom use correlates to another study where it was found that only 79.5% knew that HIV could be prevented by using condoms.^22^

Even though 12 participants improved adherence from pre-intervention to post-intervention in this study and although it was not statistically significant, there has been an improvement with the intervention of the pharmacist. This can be likened to a USA-based study where it was demonstrated that few non-compliant patients obtained an undetectable viral load prior to the pharmacists’ intervention. However, a significantly higher proportion of patients achieved an undetectable viral load after attending a pharmacist counselling clinic. In treatment experienced patients, the incorporation of a pharmacist in the HIV care team had a significant impact on patient outcomes.^25^

Pharmacist intervention resulted in an improved response in terms of side effects, from the pre-intervention to the post-intervention phases.

Pharmacists played a positive role in the way patients took their medication with regard to food. Most patients would avoid taking their medication with a high fatty meal after the intervention and took their medication correctly either with or without food. This is important because food can affect the absorption, metabolism, distribution and excretion of antiretroviral medication. Important interactions include that of Efavirenz with a high fatty meal, which should be avoided because of a reduced drug absorption.^26^

Respondents chose a variety of storage places for their antiretroviral medication, such as the bedroom, drawers in the bedroom, a drawer in the dressing table, the headboard, a box, a bag, a shoe box, an underwear drawer, on top of a wardrobe and shelves in the bedroom. This was of interest because it gave the impression that patients hid their medication. It could have been because patients did not want anyone to know their HIV status and because HIV is associated with stigma and discrimination. These individuals, if found out to be HIV positive, could face rejection by their families and communities.^27^ AIDS-related stigma and discrimination can also directly hamper the effectiveness of AIDS responses. Stigma and discrimination can directly affect the likelihood of protective behaviours.^28^

### Limitations

Firstly, this intervention study was limited to patients who were willing to participate in the study. The sample size was relatively small because it was confined to one hospital, which is a community health centre. Secondly, the reliability and validity of the participants’ self-report was unknown. Another limitation was the poor response rate after the intervention phase, that is, 12.5% of the participants abandoned the study. This could have been because most patients did not want to spend time to complete the questionnaires because they might have been aware of the time it took to complete the questionnaire in the first phase and the answers required of them, and might not have felt comfortable to repeat the process. Another reason indicated by many patients was that they wanted to collect their medication and leave the clinic quickly because of transport problems and/or the need to return to the workplace. A few patients just mentioned that they were in a hurry.

## Conclusion and recommendations

This study shows that a pharmacist-led intervention can bring about a significant improvement in the knowledge of HIV and AIDS patients regarding the HIV virus, its mode of transmission, prevention methods and antiretroviral medication use, storage as well as adherence to medication. Pharmacists play a crucial role in distributing AIDS-related information to the public and promoting the understanding of HIV and AIDS.

Pharmacists, whether in the public or private healthcare sector, should become more involved in the management of HIV infected patients and should play a pivotal role in the dissemination of HIV and AIDS information. The data from this research study could be a useful guide in the development of campaigns or programmes designed to convey accurate information about HIV and AIDS in terms of knowledge and adherence. Education based interventions may improve adherence to HAART. Interventions include patient education, counselling, health promotion, reminders and the provision of resources.
